# Bivalent genes that undergo transcriptional switching identify networks of key regulators of embryonic stem cell differentiation

**DOI:** 10.1186/s12864-020-07009-8

**Published:** 2020-11-18

**Authors:** Ah-Jung Jeon, Greg Tucker-Kellogg

**Affiliations:** 1 Department of Biological Sciences, Faculty of Science, National University of Singapore, 16 Science Drive 4, Singapore, 117558 Singapore; 2 Computational Biology Programme, Faculty of Science, National University of Singapore, 21 Lower Kent Ridge Road, Singapore, 11907 Singapore

**Keywords:** Bivalency, Embryonic stem cells, scRNA-seq, Bimodality, Chromatin state, Pseudotime, Genome regulatory network, Hidden Markov model

## Abstract

**Background:**

Bivalent promoters marked with both H3K27me3 and H3K4me3 histone modifications are characteristic of poised promoters in embryonic stem (ES) cells. The model of poised promoters postulates that bivalent chromatin in ES cells is resolved to monovalency upon differntiation. With the availability of single-cell RNA sequencing (scRNA-seq) data, subsequent switches in transcriptional state at bivalent promoters can be studied more closely.

**Results:**

We develop an approach for capturing genes undergoing transcriptional switching by detecting ‘bimodal’ gene expression patterns from scRNA-seq data. We integrate the identification of bimodal genes in ES cell differentiation with analysis of chromatin state, and identify clear cell-state dependent patterns of bimodal, bivalent genes. We show that binarization of bimodal genes can be used to identify differentially expressed genes from fractional ON/OFF proportions. In time series data from differentiating cells, we build a pseudotime approximation and use a hidden Markov model to infer gene activity switching pseudotimes, which we use to infer a regulatory network. We identify pathways of switching during differentiation, novel details of those pathway, and transcription factor coordination with downstream targets.

**Conclusions:**

Genes with expression levels too low to be informative in conventional scRNA analysis can be used to infer transcriptional switching networks that connect transcriptional activity to chromatin state. Since chromatin bivalency is a hallmark of gene promoters poised for activity, this approach provides an alternative that complements conventional scRNA-seq analysis while focusing on genes near the ON/OFF boundary of activity. This offers a novel and productive means of inferring regulatory networks from scRNA-seq data.

## Background

All levels of chromatin organization in eukaryotes are associated with covalent histone modifications that expose or constrain the genome for regulation [1, 2]. Because most transcription factors cannot bind nucleosome-bound DNA, nucleosomes must first be mobilized to expose DNA for transcription factor-driven regulation. Thus, chromatin remodeling, histone modifications, and transcriptional regulation are tightly linked.

While nucleosomes at most promoters in most cells carry either activating or repressive histone modifications, a large number of promoters in embryonic stem cells are *bivalent*, marked by both the activating H3K4me3 mark and the repressive H3K27me3 mark [3–5]. Increased bivalency is a hallmark of pluripotency, keeping developmental genes poised for commitment; bivalent promoters in embryonic stem cells are often resolved upon differentiation to either monovalent active (H3K4me3) or monovalent repressive (H3K27me3) states [5, 6].

Our understanding of transcription is being revolutionized by single-cell RNA sequencing (scRNA-seq) [7], which provides new opportunities to investigate the effects of chromatin organization on transcription [8]. However, most of what is known about transcriptional control by chromatin factors is based on population-averaged data, since single-cell technologies to interrogate chromatin state are still limited. In addition, we might expect genes whose expression is associated with changes in chromatin state to toggle between active (ON) and inactive (OFF) expression states in single cells [9], which poses special problems for integrative analysis.

One challenge is that scRNA-seq data commonly yields many genes with measured expression of exactly zero in many cells. Zeros may appear because of limited read depth in single cells, or from technical sources of zero inflation. In either case, the frequent zeros present challenges for both normalization and for detection of differential expression [10]. In most scRNA-seq analyses, genes with a high proportion of zeros are ignored and genes with high variability of non-zero values are kept.

Cell state-dependent genes in scRNA-seq data are often chosen first on the basis of high cell-to-cell variability. These Highly Variable Genes (HVG) may include genes differentially expressed in bulk transcriptomic studies, and may include genes indicative of underlying cell variability. HVG detection methods include exploiting the coefficient of variation [11] or squared coefficient of variation [12]. Some methods like BASiCS [13] require spike-in genes to estimate technical variation. Many HVG detection methods are computationally intensive and require genes to be filtered prior to the main analysis. This filtering often excludes genes with low mean expression.

Motivated by our interest in chromatin bivalency, our working model is that important stochastic expression changes occur right at the transcriptional boundary between active and inactive states [9]. Genes that undergo such transcriptional switching often include a high proportion of zeros in the expression data, or mean expression levels that are not high enough to be detected as highly variable. Therefore, we needed a new method with a conceptually different underlying principle to identify those switching genes.

To identify transcriptional switches at bivalent (including poised) promoters, we focus on genes that are sometimes silent, and other times expressed. We show that a modified Gaussian mixture model can identify genes undergoing transcriptional switches, even in the presence of the abundance of zeros common to scRNA-seq data. We refer to these as “bimodal” genes. We associate these bimodal genes with chromatin state from independent ChIP-seq studies and find that important developmental processes are enriched in bimodal genes carrying bivalent histone marks in pluripotent cells, and that bimodal bivalent genes can be used to characterize cell types.

We employ binarized gene expression for feature selection, and identify known and novel genes activated or silenced upon differentiation of embryonic stem cells to distinct cell types. We show how fractional state proportion of binarized gene expression data is a useful complement to conventional differential expression measures for many genes, and allows us to identify genes that switch activity with cell state change.

We apply a similar approach to time course differentiation data and develop a pseudotime ordering method exploiting probabilistic suffix trees of binarized data. The pseudotime ordering allows us to identify switching events using a hidden Markov model in pseudotime, when genes switch between mostly OFF and mostly ON states. We use the orchestration of these events in pseudotime to infer a regulatory network of gene switching.

## Results

**Bimodality-based variable gene detection identifies genes in ON/OFF transcriptional states**

We define expression variability as the toggle between active and inactive transcriptional states. Variable genes, therefore, are those that are active in some cells and inactive in others in a population. Across the population, we expect the variable genes to show clear separation of expression values, resulting in a bimodal distribution.

For identification of bimodal genes, we define the underlying model of gene expression as a mixed lognormal distribution with possible zero inflation (Eq. 6 in “Methods” section). We allow the Gaussian Mixture Model to have two or three centers. Even though a truly bimodal gene will only have two centers, we observed genes with three centers and decided to include the optional intermediate state, which could arise from imprecise normalization of expression values or from the existence of a true intermediate state. The component with the smallest mean corresponds to the inactive state and we expect it to occur near zero; others will appear anywhere away from – but distinctly away from – zero. Bimodal genes were selected using Hartigan’s Dip Test for non-unimodality, followed by fitting a two or three centered Gaussian Mixture Model on appropriately transformed data (see “Methods” section). Genes with *p*<0.01 and a smallest mean below 1 were selected as bimodal genes.

We applied this approach on two scRNA-seq datasets [14]. The first dataset (H1 data) uses H1 cells and their differentiated derivatives, which were FACS sorted based on known cell types, including definitive endoderm cells (DEC), endothelial cells (EC), neural progenitor cells (NPC), trophoblast-like cells (TB), and human foreskin fibroblasts (HFF). The second dataset (H9 data) uses H9 cells differentiated towards endoderm and collected at various time points (0h, 12h, 24h, 36h, 72h, and 96h) after stimulation.

The two datasets presented two different experimental groupings. In H1 data, cells were grouped based on cell types, while in H9 data, cells were grouped based on experimental time points. We applied our bimodal gene identification method to H1 data to explore differentiation signature genes and genes with poised promoters, and later to H9 data to construct and analyze a pseudotime trajectory based on bimodality.

To include unexpressed or undetected genes – which appear as a single point function at zero – in our mixture model, we added a small amount of normally distributed noise to all transformed data. In the preliminary analysis with H1 data, we varied the amount of added noise and determined the optimal standard deviation of added noise as 1×10^−5^ (Fig. S1 and Table S1, Additional File 1). Too little noise or too much added noise reduced the number of bimodal genes detected. Re-sampling showed that the small amount of added noise generated a stable and consistent identification of bimodal genes (Fig. S1, Additional File 1). After optimization of noise we identified 2610 bimodal genes from H1 data and 4943 bimodal genes from H9 data.

**Bivalency distinguishes cell type dependent bimodality in H1 cell differentiation**

In embryonic stem cell differentiation, histone bivalency (H3K4me3 and H3K27me3) marks important developmental and differentiation regulatory genes [5]. Bivalently marked genes are kept in a poised state before lineage-specific transcriptional fate is determined. Bivalent genes therefore represent genes that will potentially undergo transcriptional switching upon differentiation. Bivalent genes that show cell type-specific bimodality upon differentiation should therefore include marker genes and signature genes for differentiation.

We integrated the 2610 bimodal genes from scRNA-seq analysis of H1 data with independent ChIP-seq data of the same cell types to focus on those bimodal genes that are bivalently marked in H1 cells. This selection is meant to include those genes poised for transcription while in the undifferentiated state. This integrative analysis resulted in 644 bivalent, bimodal genes from the H1 data.

**Fig. 1:**
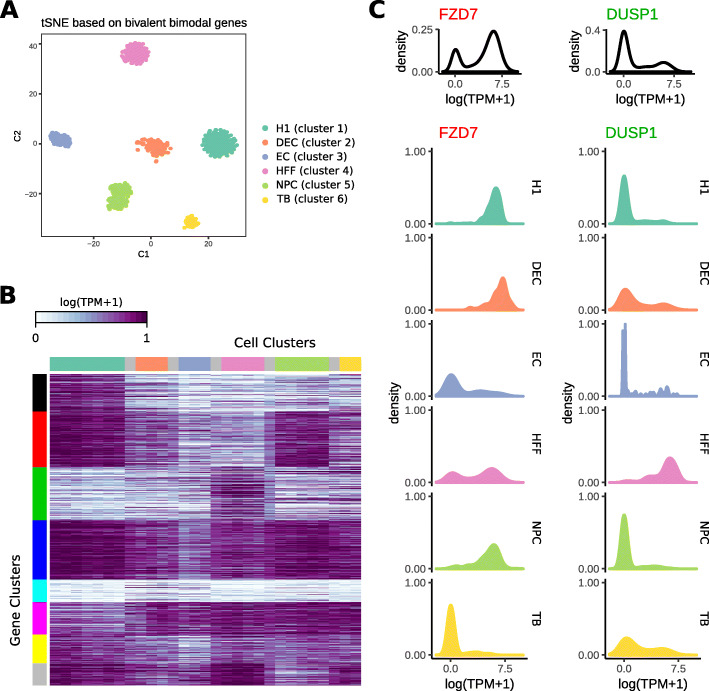
Bivalent and bimodal genes in H1 differentiation data. (**a**) t-SNE plot of bivalent and bimodal genes in H1 differentiation data. Cells were colored based on cell types, which perfectly matched the clusters. (**b**) Heatmap of fractional expression values obtained from the ratio of active cells per window. Columns represent cells and rows represent genes. Cells were ordered by cell types, split into 30 windows and colored by the cell type (Grey represents the cells in between two cell types). Genes were clustered based on the fractional expression values. (**c**) Example genes from clustering shown in (**b**). FZD7 belongs to the 2nd gene cluster (red) and DUSP1 belongs to the third gene cluster (green). Top panel shows the overall density of log(TPM+1). Bottom panel shows the density of log(TPM+1) for cells in each cell type

When cells were clustered based on the expression profiles of bivalent bimodal genes, all of the cells clustered perfectly accordingly to the experimentally determined cell type (Fig. 1a). This analysis shows that the bivalent and bimodal genes indeed show cell type-specific expression patterns. MSigDB analysis provided additional support for this interpretation, as the genes were enriched in developmental and differentiation-specific genes (Table S2, Additional File 1). In the original paper that generated the H1 data set [14], the authors used a curated list of 52 lineage-specific markers for hierarchical clustering of cell. Our method provides an unsupervised discovery of a large number (644 genes) of cell type-specific transcriptional gene switching.

In contrast to bivalent bimodal genes, when we used monovalent H3K4me3-marked bimodal genes in H1 cells for clustering, the cell type-specific clustering was much weaker (Fig. S2, Additional File 1). MSigDB analysis showed that these genes were enriched in cell-cycle regulators. This suggests that the H3K4me3-marked bimodal genes include a large number of genes whose bimodality in scRNA-seq data is not due to changes in cell identity, but due to changes in cellular state. (Table S3, Additional File 1).

**Binarization of bimodal gene expression identifies cell type-specific patterns of expression**

As active and inactive states of bimodal genes have clearly distinguishable expression values, expression values of bimodal genes can be binarized. For each bimodal gene, the local minimum expression value was taken as the binarization threshold. Cells with expression value less than the threshold were considered inactive (OFF), and cells with expression value more than that were considered active (ON).

We used the clustering obtained above to assign fractional gene activity between 0 and 1 by assigning cells to random windows within each cluster, converting the original 750 cells with binarized expression to 30 windows with fractional activity. Using these fractional expression profiles, we clustered bivalent bimodal genes into 8 groups (Fig. 1b). Visualization of fractional expression values showed much more striking patterns than the individual cells: most groups showed cell type-specific transcriptional states, especially gene clusters 1,2,3, and 4. For example, gene cluster 2 (red) showed mostly active transcription in H1 and NPC cells, and less active transcription in other cell types, while gene cluster 3 (green) showed mostly active transcription in HFF and mixed in others. Example genes and their expression profiles in each cell type are shown in Fig. 1c. FZD7 is from gene cluster 2, and it is mostly in active states in H1, DEC, and NPC cells. It forms a mixed population of both active and inactive states among HFF cells, while is mostly inactive in EC cells. DUSP1 (from gene cluster 3) is mostly active only in HFF cells. Both FZD7 and DUSP1 show overall bimodal expression profiles (top, Fig. 1c) while showing unimodality within each type of cell. These are examples of genes exhibiting cell type-specific bimodality.

**Binarization of bimodal genes allows differential expression testing based on bimodality**

**Fig. 2:**
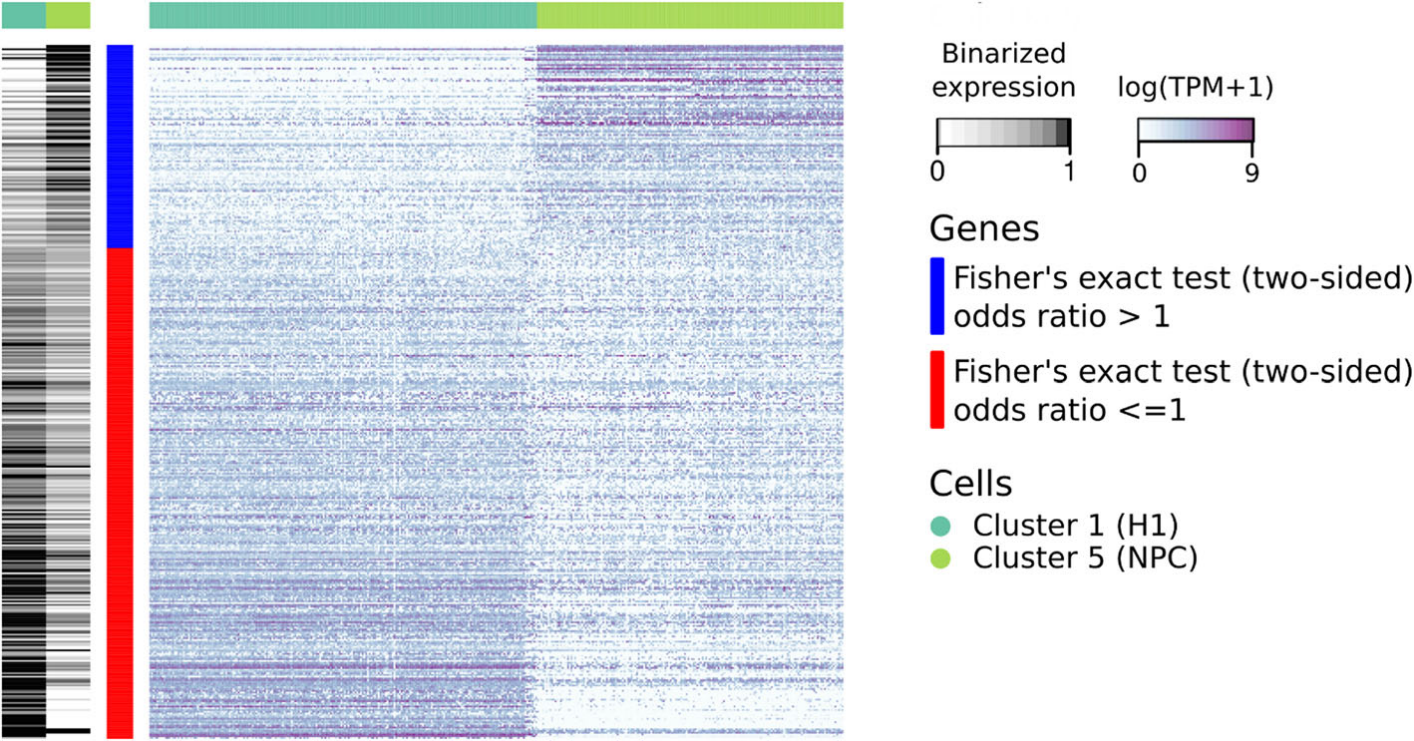
Genes that undergo transcriptional switching upon differentiating into Neural Progenitor Cells. Genes with adjusted *p*-value less than 0.05 are shown. The heatmap in greyscale (left) shows the fractional expression values across all cells in each cell-type. The heatmap on the right shows the actual log(TPM+1) expression values of the genes

To identify genes that showed transcriptional switching between cell types, we used the binarized expression values for bimodal genes. For a pair of cell types including H1 and an H1 derivative, we then used Fisher’s exact test to identify genes that showed clear changes in the proportions of active cells upon differentiation. The odds ratio was used to infer the direction of differential activity, with genes with odds ratio greater than one being upregulated as less than one being downregulated upon differentiation (Figs. 2 and S9, Additional File 1). “Upregulated” in this usage does not mean expressed at a higher magnitude, but active in a higher proportion of cells. Enrichment analysis showed that this method of differential expression switching was consistent with cell-type specificity. For example, genes that were more often inactive in H1 cells and active in NPC cells were enriched in developmental, neurogenesis, and neuron differentiation GO categories (Table S4, Additional File 1). Our bimodality-based transcriptional switch identification method successfully identifies signature genes of cell type-specific differentiation.

**Variation of information can be used to infer a pseudotime trajectory for binarized bimodal genes in time series data**

The analyses described above are valuable for discrete cell states, but many single-cell studies use cells collected over time. With the perspective that gene expression changes represent underlying changes in transcriptional state, we asked whether one could infer the underlying time order of cells in a differentiation model, and use that cell ordering to identify the timing of transcriptional switching. We used data from an endoderm differentiation study of H9 cells (H9 data) [14]. In this study, H9 cells were differentiated towards endoderm and cells were collected and sequenced at different timepoints.

We used the 4943 bimodal genes and asked whether we could use binarized bimodal genes to infer a pseudotime trajectory of cells. Unlike H1 data where a clear distinction between cell types was important to reveal marker genes, an overly clear distinction between time-dependent cell clusters in H9 data would potentially compromise pseudotime trajectory reconstruction. Therefore, we included H3K27me3 monovalent bimodal genes in addition to the H3K4me3/H3K27me3 bivalent bimodal genes. We also kept H3K27me3 monovalent-marked genes to identify genes that showed de-repression upon differentiation. A total of 1371 genes were selected.

The selected genes characterized cells at each timepoint well, with the exception of cells from 72 and 96 hours of treatment (Fig. S3a, Additional File 1), consistent with the similarity at these timepoints noted in the original paper [14]. Cells in cluster 5 and 6 were therefore combined for downstream analysis (Fig. S3b, Additional File 1).

Existing pseudotime inference methods are not designed for genes with a high proportion of zeros, as we see for bimodal genes, and not designed for binarized gene expression. We devised a new algorithm to use binarized bimodal gene expression for cell ordering. In addition, since in this data set cells were collected at known time points throughout differentiation, the order of clusters was known.

**Fig. 3:**
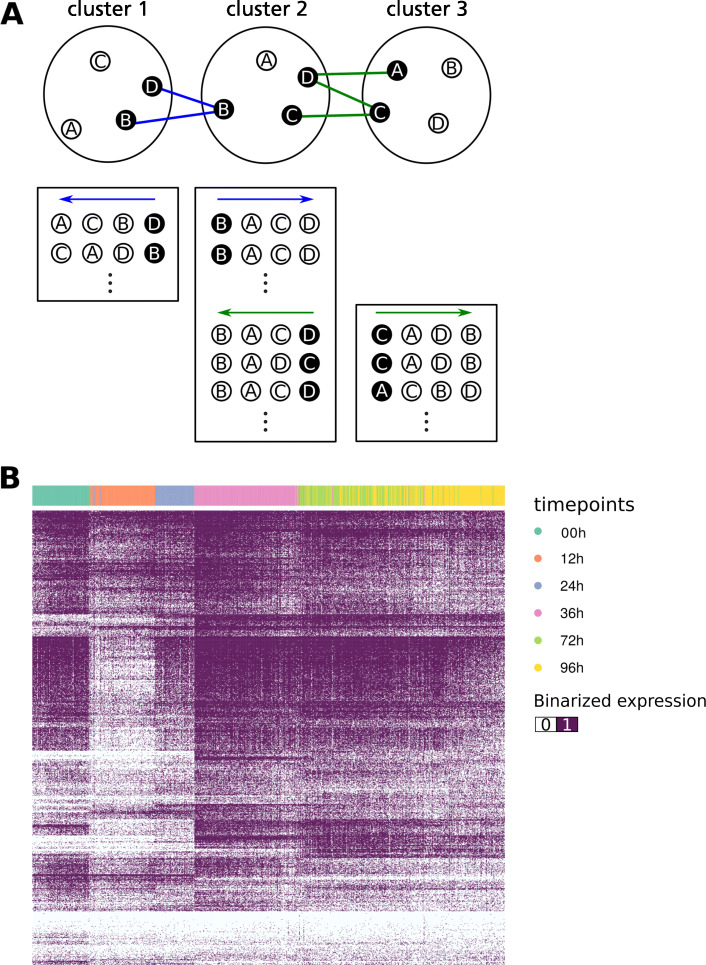
Pseudotime trajectory estimation (**a**) *Top*. Each circled letter represents a cell in a cluster. Letters highlighted in black represent the *connecting cells* that show low variation of information to cells in the adjacent cluster. Cells in clusters are unordered. *Bottom.* Cells arranged in different orders within rectangles represent the cell orderings (*sequence*) within each cluster based on the variation of information between the *connecting cells* and the rest of the cells in the cluster. Refer to the main text for more explanation. (**b**) Heatmap of log(TPM+1) expression values of bivalent and bimodal genes from H9 time-series data. Cells (columns) are arranged according to the pseudotime ordering and colored according to the experimental timepoints. Genes (rows) are ordered using hierarchical clustering

Figure 3a graphically summaries our pseudotime trajectory estimation method. Our algorithm orders clusters of cells by known time points, and then generates candidate cell orderings within each cluster. The candidate cell orderings are then used to generate a probabilistic suffix tree of all cells, from which a final pseudotime ordering is inferred. The procedure is described below.

We defined distances between cells as the variation of information (VI, a metric related to mutual information) of binarized gene expression. To order the cells within each cluster, we used the adjacent time points (clusters) as anchors (*connecting cells* in Fig. 3a). VI distances between cell pairs in adjacent clusters were calculated, and the 100 closest pairs were chosen as candidates to connect clusters. For each candidate pair, a candidate ordering was defined by VI distance from the candidate connecting cell, resulting in 100 orderings for the first and last clusters, and 200 orderings for the clusters in between.

Using the obtained set of orderings (*sequences*), we built a probabilistic suffix tree (PST) for each cluster, followed by sequence generation. If no unique sequence could be obtained, we selected the ordering with the highest probability based on the PST. We observed that all clusters generated unique sequences except for cluster 1. We believe this is because cluster 1 was formed from untreated control cells, so strictly speaking they were not on the same pseudotime trajectory as the other treated cells. This was also reflected in the t-SNE map (Fig. S3a, Additional File 1), where cluster 1 cells sat noticeably away from the rest of the clusters.

Hierarchical clustering of genes using the final cell order revealed gradual changes in the expression profiles (Fig. 3b) consistent with pseudotime inference.

**Identification of transcriptional switches during endoderm differentiation from a hidden Markov model**

**Fig. 4:**
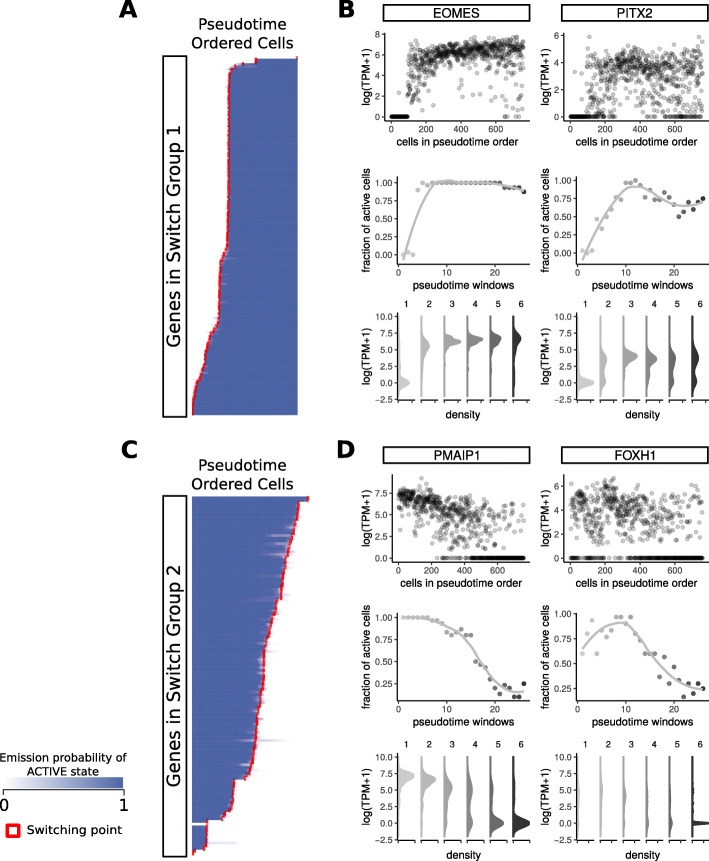
Switch group 1 and 2 genes with activated and deactivated transcriptional states Genes are grouped according to the switch groups. For switch group 1 and 2, genes are ordered within each group according to the first switching timepoints (denoted in red) in increasing order. (**a,c**) the Viterbi path of active transcriptional state for genes in switch group 1 and 2, (**b,d**) expression profiles of example genes from switch group 1 and 2. Top: log(TPM+1) expression values in pseudotime order. Middle: Fraction of active cells at each pseudotime window. Cells were split into 26 windows. Bottom: Density plot for log(TPM+1) expression values at each time-ordered cell cluster. X-axis scales are 0-0.75, 0-0.6, 0-0.6, and 0-2 respectively

To identify when transcriptional switching occurs along the reconstructed pseudotime scale, we used the Viterbi algorithm [15] to find the most likely underlying hidden transcriptional states that resulted in the sequence of observed binarized expression values. Out of 1371 genes, 1063 genes showed clear switching patterns with well defined Viterbi paths (*switching genes*). The switching points for each gene were determined based on the transition between active and inactive state paths in pseudotime-ordered cells. We classified genes into 17 switch groups, based on the initial state, final state, and the number of switches (Fig. 4, as well as Table S5 and Fig. S4, Additional File 1). Switch group 1 included genes that were switched on with a single switch from inactive initial to active final state, active final state; switch group 2 included genes that were switched off with a single switch from active initial to inactive final state. The rest of the switch groups included genes with multiple switches through endoderm differentiation.

As cells were collected at time points far enough apart to establish distinct expression profiles, most of the switches occurred near the cell cluster boundaries as shown by the vertical streaky patterns in the heatmap (Fig. S4, Additional File 1). Some genes switched sequentially, as shown by the diagonal switching patterns, especially genes inactivated late in differentiation (Fig. 4, Switch Group 2). Figure 4 shows example genes from the switch groups 1 (EOMES and PITX2) and 2 (PMAIP1 and FOXH1). Fractional expression values (middle) show gradual changes, but the density plots per cell cluster (bottom) show that expression values take distinct bimodal patterns with switch-like activation or deactivation.

**Pathway genes undergo switching during endoderm differentiation**

To highlight the underlying biological themes behind the switching genes, we computed overlaps with other gene sets in MSigDB [16, 17]. We first focused on gene sets in CP (Canonical pathways), CP:BIOCARTA (BioCarta gene sets), CP:KEGG (KEGG gene sets), and CP:REACTOME (Reactome gene sets) to evaluate the involvement of switching genes in various pathways. The top pathway overlaps included P53 signaling, TGF-beta signaling, SMAD2/3 nuclear signaling, MAPK signaling, and WNT signaling.

The switching genes involved in signaling pathways were mostly specific to one pathway (Fig. S5, Additional File 1, top right), but their enrichment does not tell us if the switching within a pathway is coherent. We visualized pathway-level switching using stairstep graphs of switching genes within each pathway over pseudotime order (Fig. S5, Additional File 1, bottom). Many of the P53 signaling pathway genes were active during the early phase of endoderm differentiation, while the majority of Wnt signaling pathway genes were active during the late stage. Most of the switching genes peaked during the mid-phase before getting switched off, indicating the involvement of these pathways in regulating endoderm differentiation.

The stairstep plot alone makes it appear that many pathways are less active late in differentiation of H9 cells to endoderm. This may be the case, but the emission probabilities for individual genes tell a richer story: many genes that were switched on remained active, while other genes either were transiently activated or switched off.

**Transcriptionally switching genes include transcription factors and their targets**

To identify potential transcription factor targets among the switching genes, we computed enrichment between the switching genes and the TFT (transcription factor targets) gene set from MSigDB [16] using a Fisher’s exact test. The results show striking enrichment for specific transcription factors in this gene set (Table 1). The top 10 hits included SP1, MAZ, E12, LEF1, NFAT, and AP4.

**Table 1:** Top 10 motif hits for switching genes from MSigDB search. 1340 genes were compared to gene sets in C3 TFT (transcription factor targets) gene sets [16]

Gene Set Name	# Genes in Overlap	FDR q-value
GGGCGGR_SP1_Q6	273	1.13×10^−64^
GGGAGGRR_MAZ_Q6	231	4.79×10^−61^
CAGGTG_E12_Q6	221	2.17×10^−48^
CTTTGT_LEF1_Q2	185	3.19×10^−43^
TGGAAA_NFAT_Q4_01	170	5.06×10^−37^
CAGCTG_AP4_Q5	148	6.78×10^−36^
TTGTTT_FOXO4_01	172	1.09×10^−33^
AACTTT_UNKNOWN	162	5.16×10^−33^
GGGTGGRR_PAX4_03	129	2.36×10^−32^
CTTTAAR_UNKNOWN	94	2.39×10^−22^

**Fig. 5:**
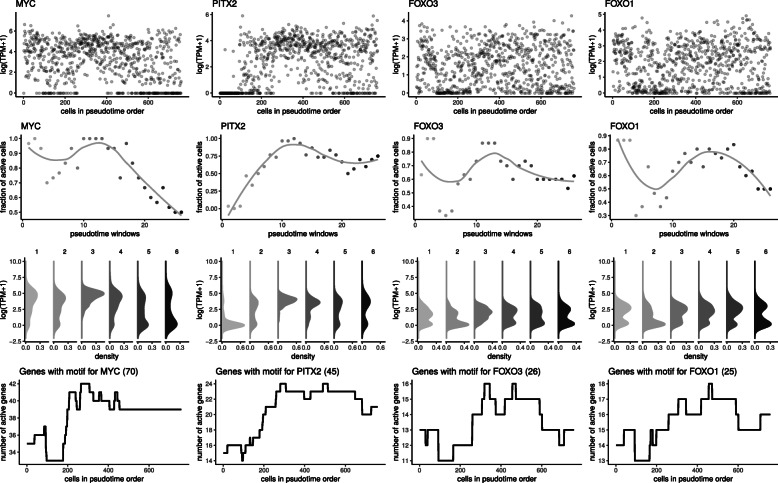
Transcription factors that undergo switching together with their downstream targets First row: Plots of log(TPM+1) expression values for transcription factors that came up in MSigDB motif search. Second row: Fraction of active cells for transcription factors that came up in MSigDB motif search. Third row: Density plot of log(TPM+1) expression values for transcription factors that came up in MSigDB motif search. Fourth row: Total number of active switching genes with transcription factor motifs

Gene regulation by transcription factors often shows a correlation between a TF and its targets. Some of the transcription factor hits were bimodal genes themselves, so we examined whether the switching pattern of a TF was recapitulated in the switching pattern of predicted targets. We compared the expression of four bimodal TFs (MYC, PITX2, FOXO3, and FOXO1) to the aggregated number of transcriptionally active TF targets that are also transcriptionally switching (Fig. 5). In each case, the aggregated number of active target genes peaked at a similar time point as the expression of the transcription factor. Some motif hits showed overlapping context to some of the earlier pathway hits. LEF1 and E12 (TCF3) came up as the top switching transcription factors, with many downstream targets also undergoing transcriptional switching. These TFs are known to be involved in canonical Wnt/beta-catenin signaling [18], which came up in the pathway search. Beta-catenin nuclear signaling-related genes peak during the mid-phase of endoderm differentiation, before deactivation at the late stage. The majority of E12 and LEF1 targets are active at similar time points as Wnt signaling pathway genes (Fig. S6, Additional File 1). This suggests that the involvement of canonical Wnt/beta-catenin signaling during endoderm differentiation may be mediated through activation of LEF1 and TCF3.

**A network of switching genes based on mutual information**

Based on the pathway genes identified from the earlier analysis, we wanted to draw a map of key regulatory genes involved in endoderm differentiation. In addition to the pathway genes, we identified *connector genes* that are closely related to the pathway genes based on variation of information. We assumed that the connector genes are the mediating regulators that help to establish a more interconnected map. With 68 switching genes that were involved in the pathways identified from the earlier pathway search, we selected the top 20 most related connector genes.

**Fig. 6:**
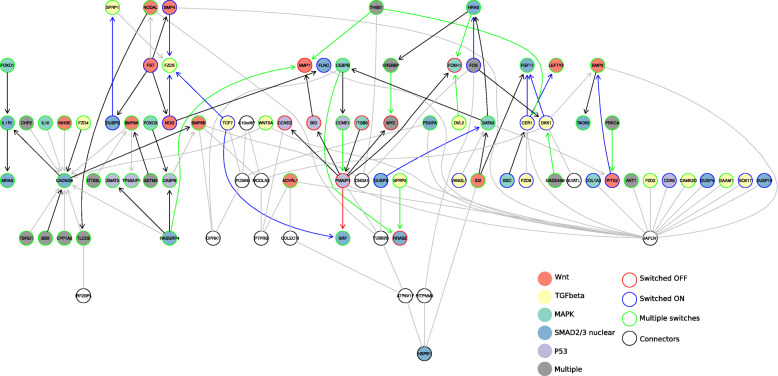
Co-switching network of genes based on mutual information Undirected edges represent genes that switch at the same time. Directed edges represent the tail gene switchedd earlier than the head gene. Grey edges represent genes that switched at similar time (less than 10 cells apart). The rest of the edge colors followed the color of the tail gene

Co-expression networks are mapped using a distance metric obtained from expression data [19], to establish significant relationships between pairs of genes. We wanted to build a similar network, but with binarized expression data. We built a network map using Algorithm For The Reconstruction Of Accurate Cellular Networks (ARACNE), which takes the mutual information matrix and returns the inferred network [20] (Fig. 6). We chose ARACNE because binarized expression data lends itself to a mutual information-based network reconstruction, and because the ARACNE algorithm used mutual information to remove uninformative edges that might otherwise complicate the inferred network.

The ARACNE network is undirected, but we assigned direction to some edges based on the switching times of the connected nodes (genes). If gene A had undergone transcriptional switching earlier in pseudotime than gene B, the edge connecting A and B was directed from A to B. This was based on the assumption that a prior event would influence later events. The network may not represent the actual gene regulatory network, but displays the co-switching relationship between key pathway genes during endoderm differentiation.

The network structure shows several interesting properties related to the earlier pathway and transcription factor analyses. Frizzled genes (FZD2, FZD4, FZD5, FZD8) and bone morphogenetic protein (BMP) genes (BMP2, BMP4, BMP7, BMP8A, BMP8B) appeared at different parts of the network, indicating their involvement in different signaling events. In the motif analysis we had found that switching genes were enriched in LEF1 and NFAT targets. From the network map, the edge connecting FZD5 and TCF7 further supports the presence of the canonical Wnt/beta-catenin signaling pathway. On the opposite side of the network map, we saw a hub of genes around HAPLN1. The presence of FZD2 (Frizzled 2), CAMK2D (Calcium/Calmodulin Dependent Protein Kinase II Delta), and DAAM1 (Dishevelled Associated Activator Of Morphogenesis 1) suggests that there is also non-canonical Wnt/Ca2+ signaling pathway present. The time-dependent differential expression of various Wnt and frizzled genes further supports the involvement of dynamic Wnt signaling events (Fig. S7, Additional File 1).

Many TGF-beta and Wnt signaling pathway related genes (yellow and red highlighted in Fig. 6) were retained in the network map. Interestingly, we noticed the presence of not only positive regulators of the pathways, but also negative regulators such as the BMP antagonist NOG (Noggin), Wnt antagonists SFRP1 (Secreted Frizzled Related Protein) and DKK1 (Dickkopf WNT Signaling Pathway Inhibitor 1), and TGFb antagonist INHBE (Inhibin Subunit Beta E). These indicate the dynamic regulation of pathways during endoderm differentiation, consistent with the findings from the stairstep plots (Fig. S6, Additional File 1).

We also noticed evidence for cross-talk between TGF-beta, Wnt, and MAPK signaling pathways based on the edges connecting genes of different pathways. Some examples are edges connecting INHBE-CACNG8-FZD4, FST-BMP4-FZD5, FST-DUSP5-SFRP1-FZD5, LEFTY2-CER1-FZD8, and DKK1-BMP2-TAOK2. TGF-beta and Wnt signaling genes also appeared interconnected together at the top left corner of the network map.

## Discussion

**On variable gene detection and differential expression testing**

Differential gene expression testing often includes an implicit assumption that larger gene expression changes are more responsible for phenotypic changes. Consistent with this model, expression counts are first normalized and a mean-variance relationship is often used as an indicator of interest, with highly variable genes (HVG) then selected based on a mean-variance threshold [21]. Variance-based methods focus on detecting changes in the spread of expression data, and are hence excellent in detecting changes in active transcriptional levels.

In this analysis, we instead model differential expression of signature genes happening across the transcriptional ON/OFF boundary, regardless of the actual magnitude of expression values. We choose this approach because the most functionally striking difference in expression is a switch from complete repression to activation, or vice versa. This is related to, but distinct from, the assumption behind variance-based methods.

Crossing the transcriptional on/off boundary, or switching between gene activation and repression, does not ensure bimodally distributed expression values. However, in this study we used data having distinct experimental groupings: either grouped according to cell types (in H1 data) or according to experimental time points (in H9 data). We assumed that signature genes distinguishing the cell groups must have formed stable transcriptional states within each cell group distinct from the originating cells. Such shifts in transcriptional states would result in bimodally distributed expression values across the cell groups, leaving a clear separation between the expression values from the on/off transcriptional states.

We also focused on the toggle between on/off transcriptional states because histone mark-driven transcriptional control mostly involves switching between repression and activation, especially in the poised promoter model of bivalency. This model is relevant for both the H1 and H9 embryonic stem cell differentiation data sets we used.

In a typical population-based transcriptomic analysis, differential expression descriptors (such as “increased/decreased expression”) describe gene expression as a process on a continuous scale of change. Differential expression analysis then takes the quantified or normalized read counts and performs statistical tests to discover quantitative changes in expression levels between experimental groups [21]. Genes identified as differentially expressed may show changes of transcription at any level, but bimodal genes take either of the ON/OFF transcriptional states. The magnitude of expression for each bimodal gene in a given cellular state is proportion of active and inactive cells, and gene expression changes are modeled as the dynamic shifts in transcriptional states. Therefore, differential expression testing is done differently for bimodal genes. Since expression values of bimodal genes can be binarized, we build contingency tables of genes in on/off transcriptional states between two cell types, and test to determine whether the proportions of on/off states are different depending on the cell types. We describe gene expression changes as dynamic shifts between active and inactive states. If a gene is increased in expression, in our analysis it is shown by increase in the proportion of cells in which the gene is active.

Bimodality-based identification of variable genes, therefore, presents a complementary method of detecting gene expression changes in scRNA-seq by focusing on the on/off transcriptional states. We are not advocating any superiority of one method over the other, but rather presenting a different method of identifying variable genes. If a choice were to be made between variance-based and bimodality-based methods, it must depend on the purpose of the experiment.

**Advantages of binarizing expression data**

Since bimodal genes present clearly distinguishable on/off transcriptional states, their expression values could be appropriately binarized, which presented several advantages for later analysis. In addition to using Fisher’s exact test for differential gene expression testing, binarized expression makes the data less susceptible to normalization accuracy and less biased towards genes with high expression counts. Binarized counts also enabled the use of mutual information-based analysis to detect patterns of co-regulatory genes. This is in contrast to many of the existing co-expression network methods based on expression correlation [19], and presents an alternative way to select co-regulated genes based on similar transcriptional switching events. Co-switching networks could reveal the genetic relationships that are possibly missed out by co-expression networks. Binarizing the expression data may appear to be prematurely simplifying the data, but the in-depth network and pathway analyses we had done shows that there are more things one could learn in terms of the global relationship between genes through binarization.

**Integrating chromatin state information into transcriptomic analysis**

We have seen that the presence of H3K4me3/H3K27me3 bivalency successfully identified cell type-specific bimodality in the differentiation datasets. We also saw that H3K4me3 monovalent bimodal genes were enriched in cell state-specific pathways such as cell cycle regulation. Our results suggest that chromatin states indeed offer valuable insight to further interrogate the transcriptomic data.

Our approach may have work especially well in differentiation studies because of the poised promoters of bivalent genes. Several studies have shown that bivalently marked genes are differentiation signature genes [5, 22]. Since bimodal genes are those that undergo transcriptional switching, bivalent and bimodal genes are the differentiation signature genes that indeed show clear cell-type dependent de-repression or de-activation. These are therefore the genes that are highly linked to lineage commitment and differentiation, and were indeed enriched with differentiation markers. A study done in 2017 investigated bulk RNA-seq data for bivalent genes for 34 tissues and primary cell lines, and also made a similar observation that bivalent genes in ES cells tend to show tissue-specific expression pattern [23].

Further studies linking between chromatin state and gene expression are currently limited by the availability of experimental methods. Various challenges include simultaneously measuring both quantities and achieving single-cell level for chromatin state data.

**On the use of binarized data for regulatory network inference**

Some of the earliest gene regulatory network reconstruction methods based on microarray expression data used Boolean networks. Technologies to infer networks have advanced tremendously since then, as have technologies to measure gene expression. However, single-cell RNA-seq data offers the opportunity to observe – zero inflation notwithstanding – if a gene is transcriptionally on or off in a given cell, and the binary state of its activity is informative. As we show here, genes that might otherwise be missed because they are not expressed at high enough *levels* may be useful to infer regulatory networks and reveal hidden components of pathway activation.

## Conclusions

Histone mark modifications are strongly associated with transcriptional regulation, but direct analysis in single cells is limited by current technology. By adopting a strategy to integrate population-level measurements of histone modifications and single-cell transcriptomics, we identified transcriptional switching patterns of bivalent genes in pluripotent cells associated with cell type upon differentiation. The technical strategy, using a mixture model-based method to identify genes with multimodal on/off expression patterns across cell types, is amenable to both pairwise comparisons and time course scRNA-seq experiments.

Binarizing transcript expression levels facilitates analysis of transcriptional state, and supports novel analysis analogous to conventional differential expression as well as novel pseudotime inference, estimate of switching points in pseudotime, and gene regulatory network reconstruction.

## Methods

All analysis was performed in R (version 3.5.1, [24]) and most plots were generated using the R package *ggplot2* [25].

**Data**

ChIP-seq files for H1 cells and H1 cells differentiated by treatment with BMP4 – into mesendoderm, trophoblast and neural progenitor cells were downloaded from the UCSD Human Reference Epigenome Mapping Project (GSE16256, [26])

scRNA-seq files for baseline and differentiated H1 and H9 cells were downloaded from GEO Series GSE75748 [14].

**Normalization**

For normalization, we used Transcripts per kilobase million (TPM) to scale both datasets. Transcript lengths were based on the GRCh37 genome build from the Ensembl database through biomaRt [27]. We compared different methods of normalization, and found that unnormalized data and data normalized using SCnorm both showed clearly distinct populations of zeros (Fig. S8, Additional File 1). SCnorm normalized data also inferred many more bimodal genes than TPM normalized data. While most bimodal genes (92%) from TPM normalized data were also identified using SCnorm normalized data, read count-based data, regardless of normalization, showed a distinct gap between zeros and the lowest non-zero expression values (Fig. S8, Additional File 1). This is unsurprising, but the gap increased the apparent detection of bimodal genes. TPM-normalized data is effectively continuous on the non-negative real line, and hence allowed detection of bimodality without this artifact. Normalized data were then transformed by log(TPM+1), and referred to as logTPM.

The two methods of normalization were compared based on the number of bimodal genes detected in H9 data, with the threshold of dip test *p*<0.01.

**Model description** Let *Y*_*g*_ denote the vector of observed gene expression values for a gene in a population of cells. Given a population of cells, the gene may be in active or inactive states in different cells. We define inactive (OFF) and active (ON) transcriptional states, *X*_*OFF*_ and *X*_*ON*_, to denote the gene expression model of one gene based on the bimodal nature of transcription. We assume that the log of normalized gene expression values in state *i* follows a Gaussian distribution with a mean of *μ*_*i*_ and standard deviation of *σ*_*i*_.

1 $$\begin{array}{*{20}l} Y_{g}=X_{OFF}+X_{ON} \end{array} $$

2 $$\begin{array}{*{20}l} ln(X_{i}) \sim N\left(\mu_{i}, \sigma_{i}^{2}\right) \end{array} $$

We take the threshold of 1 for mean gene expression values to distinguish the two states.

3 $$  0 \le \mu_{OFF} <1 \leq \mu_{ON}  $$

Due to the nature of scRNA-seq, we add in in zero inflation component to the model, yielding a sum of two lognormal distributions for the data plus the zero inflation.

4 $$\begin{array}{*{20}l} Y_{g} & =\delta(0)+X_{OFF}+X_{ON} \end{array} $$

5 $$\begin{array}{*{20}l} ln(Y_{g}+1) & =\delta(0)+N\left({\mu}^{\prime}_{OFF}, \sigma_{OFF}^{2}\right)+N\left({\mu}^{\prime}_{ON}, \sigma_{ON}^{2}\right) \end{array} $$

We add a small amount of normally distributed noise after log transformation and model the result as a Gaussian mixture.

6 $$ {}\begin{aligned} ln&\left(Y_{g}+1\right)+N\left(0,\epsilon^{2}\right)=N\left(0,\epsilon^{2}\right)+N\left({\mu}^{\prime}_{OFF}, \sigma_{OFF}^{2}+\epsilon^{2}\right)\\&+N\left({\mu}^{\prime}_{ON}, \sigma_{ON}^{2}+\epsilon^{2}\right) \end{aligned}  $$

The amount of noise *ε*=1×10^−5^ was optimized over a wide range of values choosing the value that resulted in clear single maximum number of bimodal genes for the given data set (see “Results” section).

**Bimodal gene identification and estimation of parameters**

We first used Hartigan’s dip test [28] to test for unimodality of logTPM data using the R package diptest (version 0.75-7, [29]). Next, we fit Gaussian mixture models with 2 or 3 centers using the R package Mixtools (version 1.1.0, [30]). During optimization of the method, we considered bimodal genes as those with *p*<0.01 from the Dip test, and with *μ*_1_<1, and *λ*_1_<0.6 from mixture model fitting.

To determine the optimal level of noise to be added, normally distributed added noise was tested over the range *σ*∈[0,1]. Each run was evaluated based on the number of bimodal genes detected in H1 data, using the criteria described above. Bimodal gene selection criteria was adjusted in the final analysis to use only *p*<0.01 for the dip test.

All scripts are available from https://github.com/GTK-lab/modalv.

**Cell clustering**

For clustering of cells, we used t-Distributed Stochastic Neighbor Embedding (t-SNE) [31, 32] implemented in R (Rtsne, [33]).

**Signature analysis**

Web-based signature analysis was performed using the MSigDB “Investigate Gene Sets” tool ([17], available from http://software.broadinstitute.org/gsea/msigdb/annotate.jsp) at the Broad Institute, with default parameters. Genes were compared to C2 (curated gene sets) category, C3 (motif gene sets), and C5 (Gene Ontology gene sets) gene sets [16].

**Pseudotime trajectory estimation**

Matrix of Mutual Information and variation of information between cells were calculated using the R package *infotheo* [34]. The *PST* package was used to build probabilistic suffix trees [35].

**Network analysis**

Co-switching gene networks were generated using the R package *minet* [36] followed by directed edge setting and annotation as described above.

## Supplementary information

**Additional file 1** Additional File 1: Supplementary Figures S1–S9 and Supplementary Tables S1–S5.

## Data Availability

ChIP-seq data was obtained from GEO at GSE16256, [26]. scRNA-seq data was from GSE75748 [14]. Code for this manuscript is at https://github.com/GTK-lab/modalv.
