# Supplementary material for: Bivalent genes that undergo transcriptional switching identify networks of key regulators of embryonic stem cell differentiation
Source: BMC Genomics. 2020 Nov 18;21(Suppl 10):614. doi: 10.1186/s12864-020-07009-8 (PMC7677846; doi:10.1186/s12864-020-07009-8)
Supplement: Supplementary file 1 — Additional file 1 Additional File 1: Supplementary Figures S1–S9 and Supplementary Tables S1–S5. [file 12864_2020_7009_MOESM1_ESM.pdf]

SUPPLEMENTAL MATERIALS

Bivalent genes that undergo transcriptional switching identify networks of key regulators of embryonic stem cell differentiation

Ah-Jung Jeon[1] and Greg Tucker-Kellogg[1,2*]

[*]Correspondence:
dbsgtk@nus.edu.sg
[1]Department of Biological Sciences, Faculty of Science, National University of Singapore, Singapore, SG
Full list of author information is available at the end of the article

Supplemental Figures

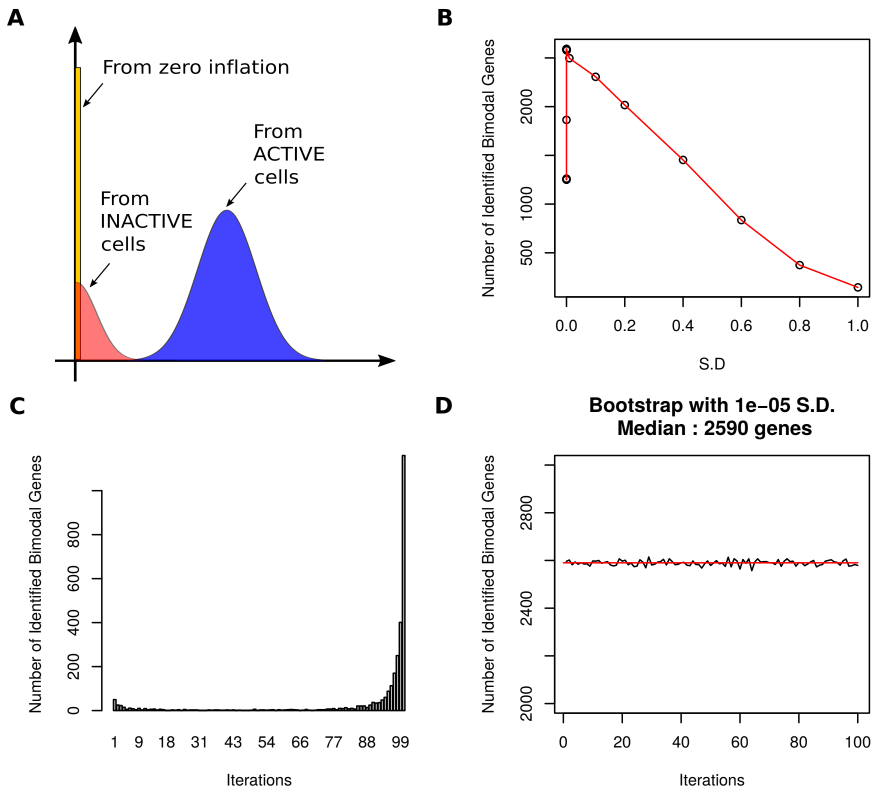

Figure S1 Schematic and noise optimization strategy. (Left) number of bimodal genes inferred with different levels of added noise. Complete list in Table S1. (Middle) Consistency of bimodal gene inference from 100 iterations using added noise of $\sigma = 1 \times 10^{-5}$. (Right) Total number of identified bimodal genes in 100 iterations using added noise of $\sigma = 1 \times 10^{-5}$.

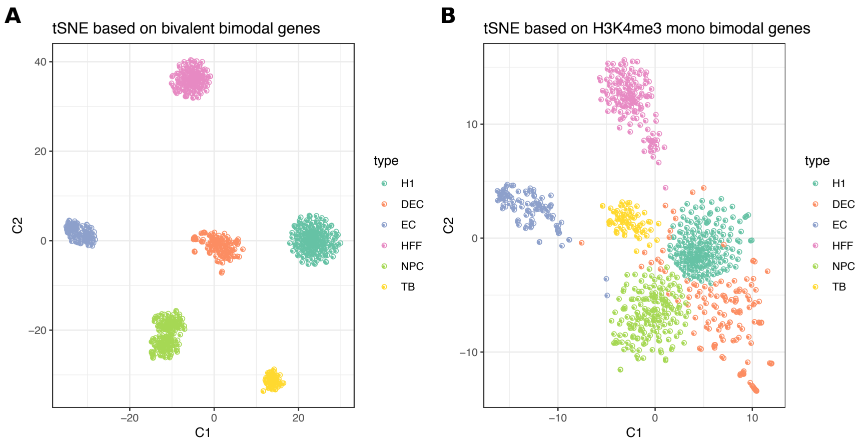

Figure S2 Clustering of H1 differentiation data based on bimodal genes in different chromatin states. (A) Clustering using bivalent and bimodal genes, colored based on cell types. (B) Clustering using H3K4me3 monovalent and bimodal genes, and colored based on cell types.

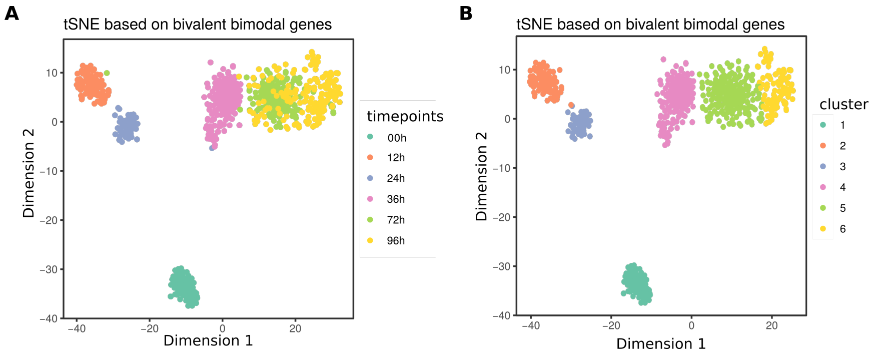

Figure S3 t-SNE plot of bivalent and bimodal genes in H9 time-series data Cells were colored based on (A) experimental timepoints, and (B) generated clusters.

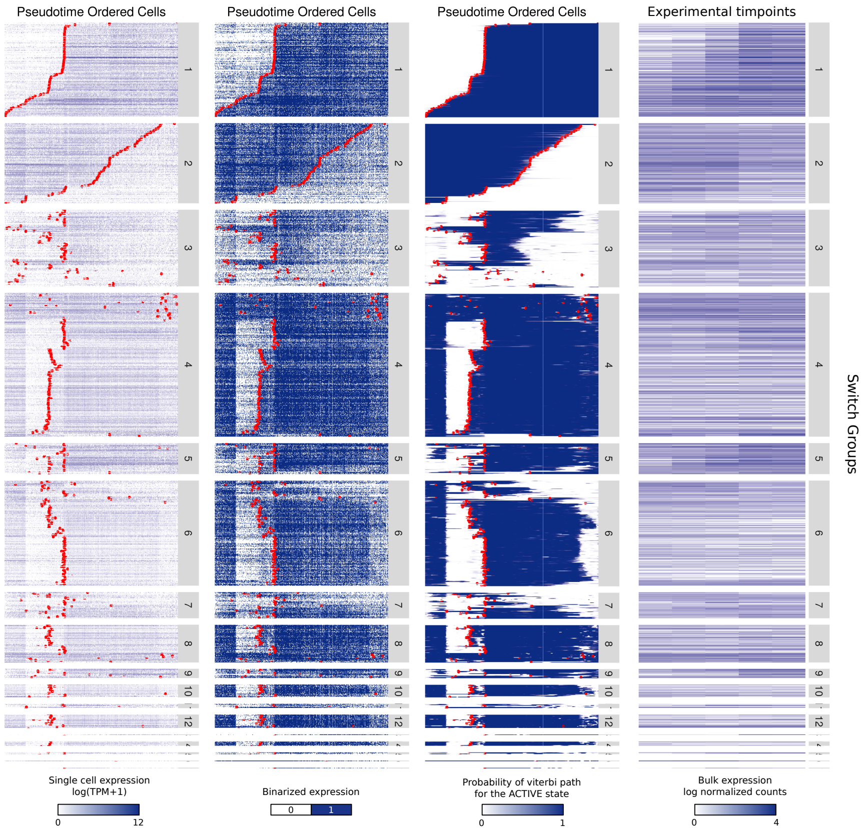

Figure S4 Hidden Markov model identification of transcriptional state switches in pseudotime Genes are shown in rows while pseudotime ordered cells are shown in columns. Genes are grouped according to the switch groups (Table S5). For switch group 1 and 2, genes are ordered within each group according to the first switching timepoints (denoted in red) in increasing order. In the rest of the switch groups, genes are ordered based on hierarchical clustering for visualisation purposes and only the first switching points (except undifferentiated cells) are denoted in red. First panel shows the log(TPM+1) expression values, second panel shows the emission probabilities of the Active transcriptional state, third panel shows the probability of Viterbi path for the Active transcriptional state, and the fourth panel shows the expression profiles of the same genes in bulk RNA-seq. Cells were collected at 5 different timepoints in the bulk RNA-seq.

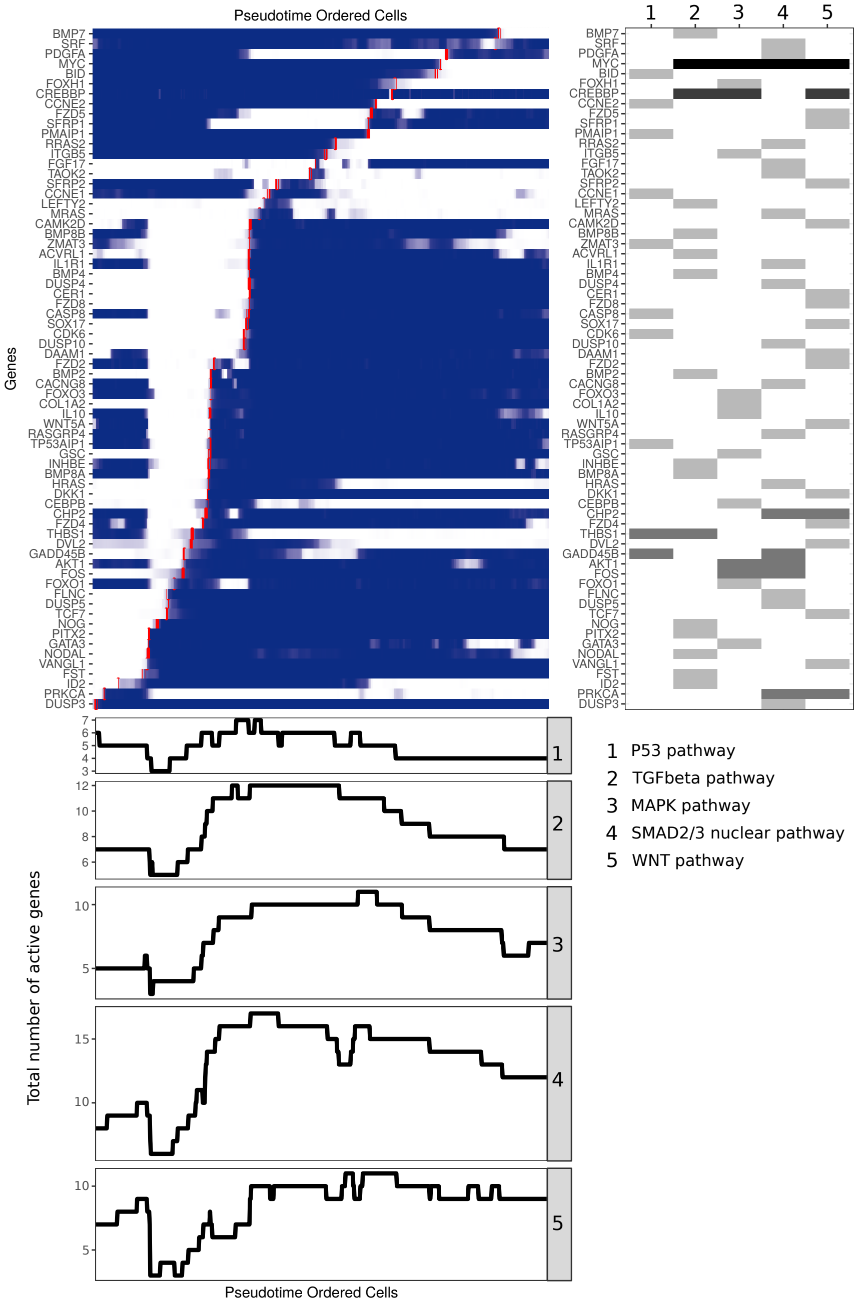

Figure S5 Switching genes involved in key regulatory pathways Top left: heatmap of emission probabilities of active states. Switching points are denoted in red. Top right: overlap between switching genes and pathway genes. The darker the shade, the gene involved in more pathways. Bottom: total number of pathway genes that are active in each cell, arranged in the pseudotime order.

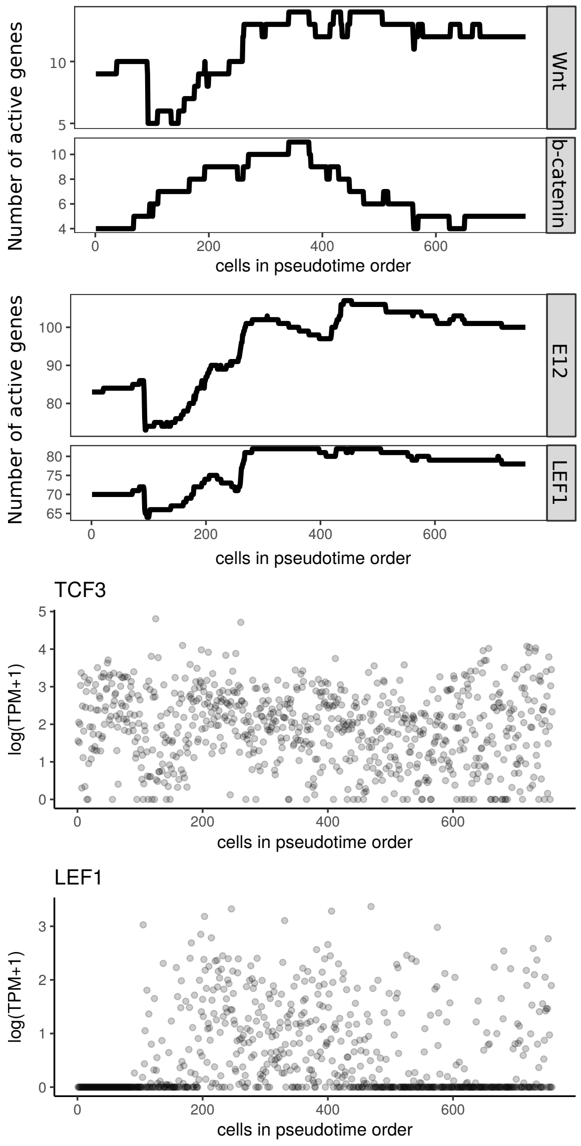

Figure S6 Canonical Wnt pathway related genes that undergo transcriptional switching First row: Total number of active switching genes that overlap with Wnt pathway genes and nuclear beta-catenin signalling pathway genes in each cell, arranged in pseudotime order. Second row: Total number of active switching genes that contain motifs for E12 and LEF1 transcription factors in each cell, arranged in pseudotime order. Third row: expression profile of TCF3 gene in log(TPM+1). Fourth row: expression profile of LEF1 gene in log(TPM+1)

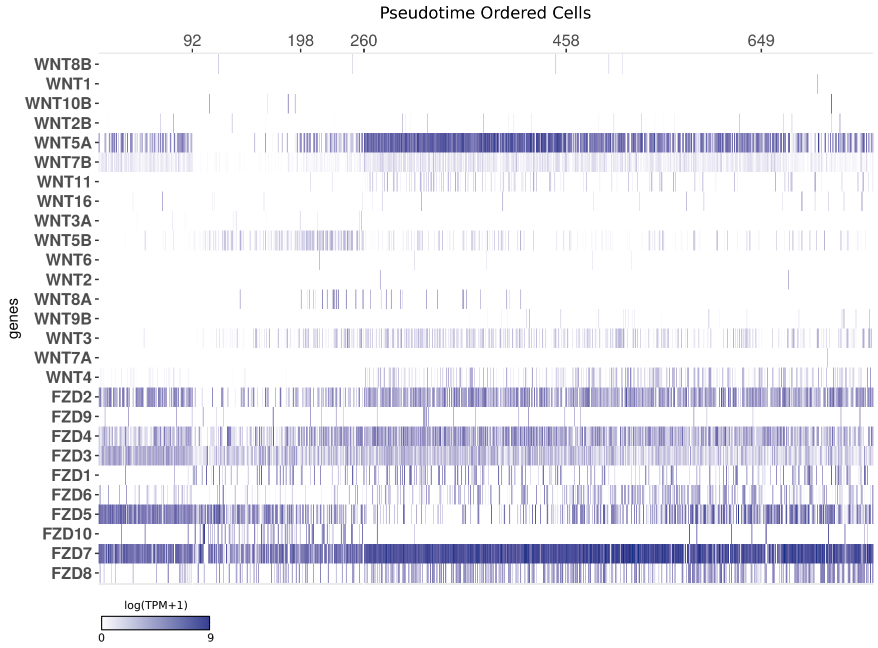

Figure S7 Expression profile of Wnt and Frizzled genes in log(TPM+1)

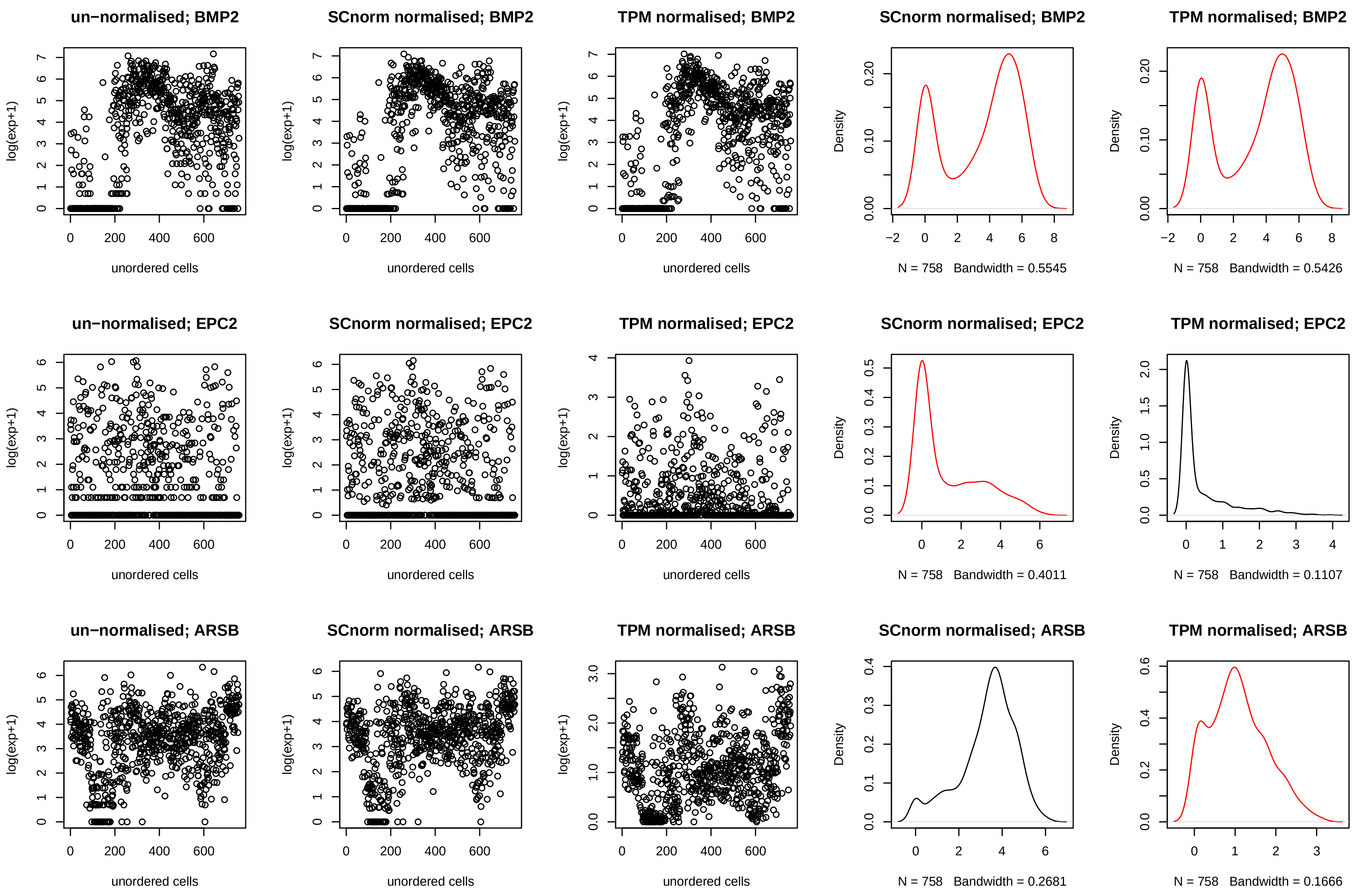

Figure S8 Comparison of different normalization methods. Three columns from the left show plots of log(TPM) expression values for all cells in H9 data. Last two columns show density plot of expression values. Plots in red shows the distributions selected as bimodal.

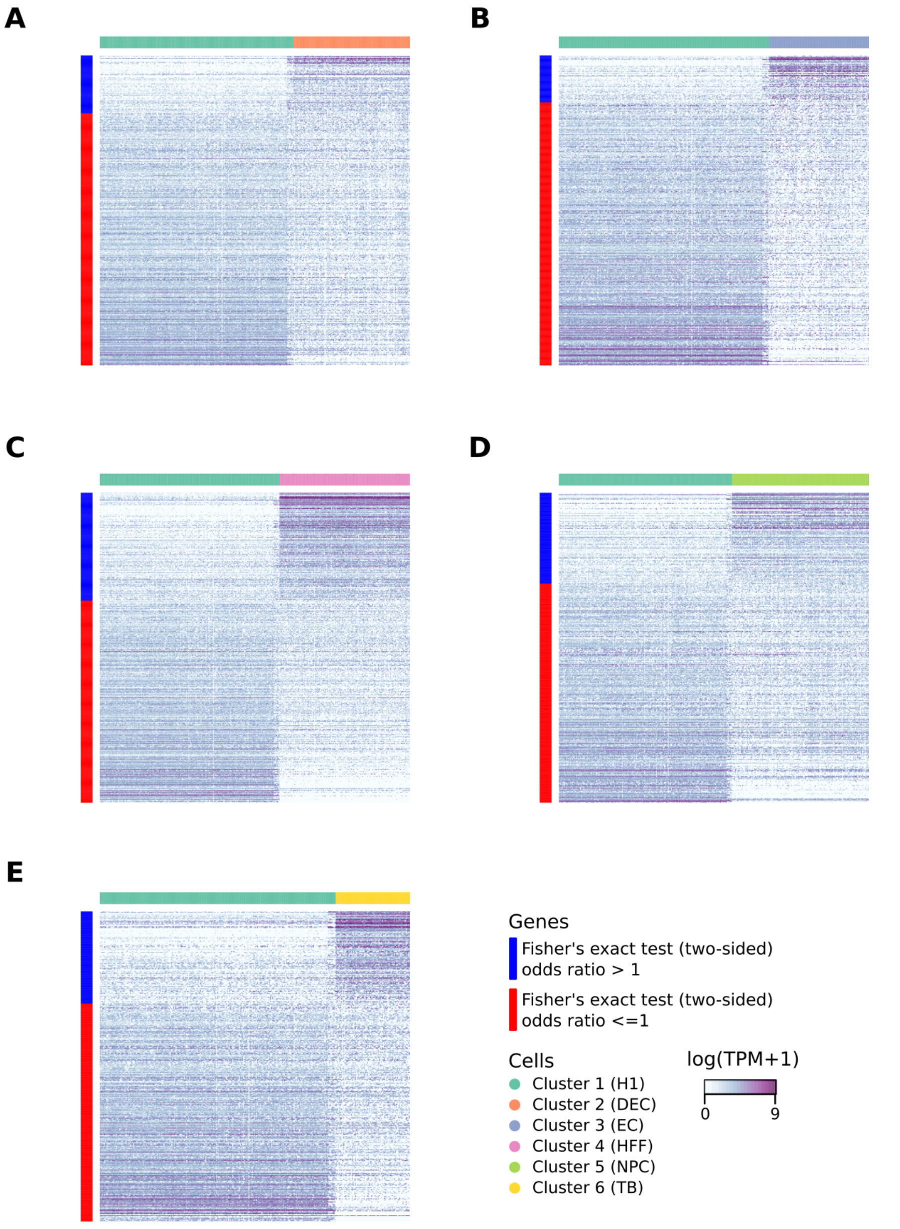

Figure S9 Genes that undergo transcriptional switching upon differentiation (A) H1 vs DEC, (B) H1 vs EC, (C) H1 vs HFF, (D) H1 vs NPC, (E) H1 vs TB

Supplemental Tables

Table S1 Values of standard deviation (S.D.) used for optimisation of noise and the number of bimodal genes detected. S.D. selected for downstream analysis is highlighted in bold.

S.D.	# bimodal genes
0	1250
1×10^{-10}	1261
1×10^{-9}	1252
1×10^{-8}	1864
1×10^{-7}	2586
1×10^{-6}	2588
1×10^{-5}	**2593**
1×10^{-4}	2579
1×10^{-3}	2579
0.01	2497
0.1	2306
0.2	2016
0.4	1453
0.6	836
0.8	374
1.0	145

Table S2 GSA analysis of bivalent bimodal genes against the C5 (Gene Ontology) collection from MSigDB. Gene set names are formatted using spaces instead of underscores for improved table readability.

**Gene Set Name**	**FDR q-value**
GO TISSUE DEVELOPMENT	3.76×10^{-41}
GO REGULATION OF CELL DIFFERENTIATION	9.69×10^{-40}
GO REGULATION OF CELL PROLIFERATION	1.92×10^{-37}
GO REGULATION OF MULTICELLULAR ORGANISIMAL DEVELOPMENT	2.4×10^{-36}
GO REGULATION OF PROTEIN MODIFICATION ON PROCESS	7.77×10^{-35}
GO NEGATIVE REGULATION OF GENE EXPRESSION	9.78×10^{-32}
GO POSITIVE REGULATION OF GENE EXPRESSION	1.31×10^{-31}
GO MACROMOLECULAR COMPLEX BINDING	4.43×10^{-31}
GO NEUROGENESIS	4.63×10^{-31}
GO REGULATION OF TRANSCRIPTION FROM RN RNA POLYMERASE II PROMOTER	1.01×10^{-30}

Table S3 GSA analysis of H3K4me3 monovalent and bimodal genes against the C5 (Gene Ontology) collection from MSigDB.

**Gene Set Name**	**FDR q-value**
GO_RNA_BINDING	3.48×10^{-125}
GO_CELL_CYCLE	5.12×10^{-119}
GO_CELL_CYCLE_PROCESS	2.33×10^{-110}
GO_MITOTIC_CELL_CYCLE	2.7×10^{-101}
GO_CHROMOSOME	5.06×10^{-101}
GO_MITOCHONDRION	5.06×10^{-101}
GO_POLY_A_RNA_BINDING	8.16×10^{-101}
GO_CELLULAR_RESPONSE_TO_STRESS	1.79×10^{-94}
GO_NUCLEOLUS	4.38×10^{-89}
GO_CHROMOSOME_ORGANIZATION	2.48×10^{-86}

Table S4 GSA analysis of genes that were activated upon differentiation into NPC against C5 collection

Gene Set Name	FDR q-value
GO_NEUROGENESIS	1.54×10^{-17}
GO_REGULATION_OF_MULTICELLULAR_ORGANISMAL_DEVELOPMENT	1.18×10^{-12}
GO_REGULATION_OF_CELL_DEVELOPMENT	1.18×10^{-12}
GO_REGULATION_OF_NEURON_DIFFERENTIATION	3×10^{-12}
GO_REGULATION_OF_CELL_DIFFERENTIATION	3.99×10^{-12}
GO_TISSUE_DEVELOPMENT	4.86×10^{-12}
GO_CELL_DEVELOPMENT	1.12×10^{-11}
GO_REGULATION_OF_NERVOUS_SYSTEM_DEVELOPMENT	1.41×10^{-11}
GO_HEAD_DEVELOPMENT	6.92×10^{-11}
GO_SENSORY_ORGAN_DEVELOPMENT	6.92×10^{-11}

Table S5 Switch groups of genes *start* indicates the starting transcriptional state of the gene. *end* indicates the final transcriptional state of the gene. *switches_on* and *switches_off* indicates the number of switches along the Viterbi path from off to on or off to on, respectively; *frequency* indicates the number of genes in each switch group.

switch_group	start	end	switches_on	switches_off	frequency
1	FALSE	TRUE	1	0	157
2	TRUE	FALSE	0	1	133
3	FALSE	FALSE	1	1	127
4	TRUE	TRUE	1	1	237
5	FALSE	TRUE	2	1	51
6	TRUE	FALSE	1	2	175
7	FALSE	FALSE	2	2	43
8	TRUE	TRUE	2	2	62
9	FALSE	TRUE	3	2	15
10	TRUE	FALSE	2	3	21
11	FALSE	FALSE	3	3	7
12	TRUE	TRUE	3	3	22
13	FALSE	TRUE	4	3	1
14	TRUE	FALSE	3	4	7
15	FALSE	FALSE	4	4	3
16	TRUE	TRUE	3	4	1
17	FALSE	FALSE	6	6	1

Author details

[1]Department of Biological Sciences, Faculty of Science, National University of Singapore, Singapore, SG. [2]Computational Biology Programme, Faculty of Science, National University of Singapre, Singapore, SG.

References
